# Characterization of a recombinant Newcastle disease virus expressing the glycoprotein of bovine ephemeral fever virus

**DOI:** 10.1007/s00705-016-3078-2

**Published:** 2016-10-18

**Authors:** Minmin Zhang, Jinying Ge, Zhiyuan Wen, Weiye Chen, Xijun Wang, Renqiang Liu, Zhigao Bu

**Affiliations:** State Key Laboratory of Veterinary Biotechnology, Harbin Veterinary Research Institute, Chinese Academy of Agricultural Sciences, 427 Maduan Street, Harbin, 150001 People’s Republic of China

## Abstract

Bovine ephemeral fever (BEF) is caused by the arthropod-borne bovine ephemeral fever virus (BEFV), which is a member of the family *Rhabdoviridae* and the genus *Ephemerovirus*. BEFV causes an acute febrile infection in cattle and water buffalo. In this study, a recombinant Newcastle disease virus (NDV) expressing the glycoprotein (G) of BEFV (rL-BEFV-G) was constructed, and its biological characteristics *in vitro* and *in vivo*, pathogenicity, and immune response in mice and cattle were evaluated. BEFV G enabled NDV to spread from cell to cell. rL-BEFV-G remained nonvirulent in poultry and mice compared with vector LaSota virus. rL-BEFV-G triggered a high titer of neutralizing antibodies against BEFV in mice and cattle. These results suggest that rL-BEFV-G might be a suitable candidate vaccine against BEF.

## Introduction

Bovine ephemeral fever virus (BEFV) is an arthropod-borne rhabdovirus that belongs to the genus *Ephemerovirus* of the family *Rhabdoviridae* [29] and causes an acute febrile infection in cattle and water buffalo [40]. The family *Rhabdoviridae* includes members of the genera *Lyssavirus* (e.g., rabies virus), *Vesiculovirus* (e.g., vesicular stomatitis virus), and *Ephemerovirus* (e.g., BEFV) and 10 other genera (e.g., fish rhabdoviruses) [7, 29]. BEF occurs mainly in tropical and subtropical regions of Africa, Asia, Australia and the Middle East [17]. It is commonly known as ephemeral fever or 3-day stiffness sickness because of the immobilization of infected animals for 3–5 days following the height of viremia and fever [2, 6]. Although recovery may be complete, mortality occurs in 2 %–3 % of cases, and a permanent drop in milk production in cows and reduced fertility in bulls often occurs, resulting in heavy economic losses [6].

The BEFV G protein is the virion envelope glycoprotein, which serves as a protective antigen [17, 20, 44]. As in other rhabdoviruses, glycoprotein G is highly immunogenic and is the target of neutralizing antibodies [13, 20, 23, 25, 36, 41]. Rhabdovirus G plays crucial roles in attachment, fusion and entry into host cells [10, 11, 26, 33, 34]. BEFV vaccines have been tested, including live attenuated virus followed by inactivated virus [19], using BEFV G as an antigen [36]. Live-vector vaccines employing a vaccinia virus vector or a South African vaccine strain of lumpy skin disease virus for expression of BEFV G have been reported [20, 41].

Newcastle disease virus (NDV) has been used in vaccine vectors for research on the characteristics of oncolytic and foreign antigens [3, 8, 12, 13, 38, 42, 43]. The NDV genome is simple, well characterized, and easy to proliferate in chicken embryos for vaccine production. NDV induces mucosal and cellular immunity [18, 32] and has been actively developed and used for the control of human and animal diseases in recent years [4, 5, 8, 9, 12, 14–16, 18, 22, 24, 37]. In this study, we used the attenuated NDV strain LaSota reverse genetics system to construct recombinant NDV expressing BEFV G (rL-BEFV-G) and evaluated its biological characteristics and immunogenicity.

## Materials and methods

### Cells and virus

Baby hamster kidney (BHK-21) and Madin–Darby bovine kidney (MDBK) cells were grown in Dulbecco’s modified Eagle’s medium containing 5 % fetal bovine serum. NDV LaSota as a vector virus was rescued from the genomic cDNA of the NDV LaSota vaccine strain (GenBank accession no. AY845400.2) with additional help from MVA-T7 as reported previously [21, 27]. The recombinant NDV strain rLaSota was grown and titrated in 10-day-old specific-pathogen-free (SPF) embryonated chicken eggs by allantoic cavity inoculation. Wild-type BEFV was grown in BHK-21 cells as described previously [39].

### Rescue of recombinant virus

pBR322 containing NDV LaSota genomic cDNA has been described previously [12]. The open reading frame (ORF) of the G gene from BEFV (GenBank accession no. JX564640.1) was produced by reverse transcription (RT)-PCR. BEFV was grown for 72 h in BHK-21 cells, with an inoculation dose of 0.01 times the 50 % tissue culture infective dose (TCID_50_) per cell. The supernatant was harvested, and BEFV genomic RNA was extracted using a Total RNA Extraction Kit (Omega, Norcross, GA, USA). The G gene was amplified by RT-PCR using the following primer pair: 5′-GACT**GTTTAAAC** TTAAGAAAAAATACGGGTAGAAGTCTG*GCCACC*atgttcaaggtcctcataattacc-3′ and 5′-GACT**GTTTAAAC**ttaatgatcaaagaatctatc-3′, in which the gene end and gene start sequences of NDV (underlined), an optimal Kozak sequence (italics), and PmeI restriction sites (bold) were introduced. The amplified BEFV G gene was sequenced and inserted into the LaSota genomic cDNA between the P and M genes. The resultant plasmid (designated as pLa-BEFV-G) was used for virus rescue as described previously [12]. The resultant recombinant virus was designated as rL-BEFV-G.

### Immunofluorescence and western blotting

BHK-21 cells were infected with rLaSota or rL-BEFV-G at MOI 1. After 24 h, the total cellular proteins were extracted with lysis buffer (1 % Nonidet P-40, 0.4 % deoxycholate, 50 mM Tris-HCl [pH 8], 62.5 mM EDTA) on ice for 5 min, and collected in 1.5-ml Eppendorf tubes, followed by centrifugation for 2 min at 15,000 × *g*. The supernatant was stored at −70 °C until used for western blotting. Western blotting was performed as described previously [12], except the primary antibody was anti-BEFV serum from mice and goat anti-mouse IgG F(ab′)_2_-peroxidase antibody (Sigma, St. Louis, MO, USA). The primary NDV antibody was produced in a chicken.

For confocal assay, BHK-21 cells were plated on coverslips in 35-mm-diameter dishes and infected with rLaSota or rL-BEFV-G at an MOI of 0.01. The experimental procedure was performed as described previously [17], except that the primary antibody was mouse serum against BEFV and FITC-conjugated goat anti-mouse antibody (Sigma) or tetramethylrhodamine (TRITC)-conjugated rabbit anti-chicken antibody (Sigma). Finally, cells were analyzed using a fluorescence or confocal laser microscope. Images were acquired using a Zeiss Axioskop microscope (Thornwood, NY, USA) that was equipped for epifluorescence with a Sensys charge-coupled device camera (Photometrics, Tucson, AZ, USA) and IPLab software (Scanalytics, Vienna, VA, USA).

### Growth in chick embryo and MDBK cells

To compare the growth kinetics in SPF chicken embryonated eggs, the rL-BEFV-G and parental strain rLaSota were inoculated into the allantoic cavity of 10-day-old embryonated chicken eggs at 10^4^ times the 50 % egg infective dose (EID_50_) in a volume of 100 μl. At 24, 48, 72 and 96 h, six chick embryos were randomly picked and allantoic fluid was used to measure the EID_50_. Monolayers of MDBK cells were infected with either rLaSota or rL-BEFV-G at an MOI of 0.01. After replacement of the medium with fresh medium, the infected cells were incubated at 37 °C in the absence or presence of TPCK trypsin (1 μg/ml). At 24, 48, 72, 96, and 120 h, the samples were collected. The virus was titrated on MDBK cells.

### Pathogenicity in poultry and mice

The intracerebral pathogenicity index (ICPI), intravenous pathogenicity index (IVPI), and mean death time (MDT) in chicken embryos were determined using the method recommended by the Office International Des Epizooties (OIE). To assess the pathogenicity of recombinant viruses in mice, 4-week-old female mice (BALB/c) (Vital River, Beijing, China) were inoculated intramuscularly (n = 10) and intracerebrally (n = 10) with rL-BEFV-G at 10^7^ TCID_50_ (30 or 100 μl). At 5 days after inoculation, tissues were collected and homogenized from five mice of each group. Viral titers in tissue were tested by indirect immunofluorescence assay (IFA) as described previously [13] and RT-PCR. The remaining 10 mice were observed daily for 2 weeks for signs of disease, weight loss, or death.

### Immunization studies in mice

Forty 4-week-old female BALB/c mice were divided randomly into four groups, and the groups were named rL-BEFV-G, rLaSota, inactivated BEFV vaccine (Weike Biotechnology, China), and phosphate-buffered saline (PBS). Ten mice in the rL-BEFV-G group were immunized with rL-BEFV-G by intramuscular injection (100 μl, 10^7^ TCID_50_). Ten mice in the rL-BEFV-G group were immunized with inactivated BEFV vaccine by intramuscular injection (100 μl, 10^5^ TCID_50_). An equal number of mice were inoculated intramuscularly with rLaSota (100 μl, 10^7^ TCID_50_). Ten mice were mock-infected with PBS (100 μl). Booster immunization was performed at 3 weeks after primary immunization. Blood samples were collected every week.

### Immunization studies in cattle

Eight Holstein calves that were seronegative for BEFV were injected intramuscularly with 4 ml of allantoic fluid for rL-BEFV-G (2 × 10^7^ TCID_50_/ml) or 4 ml of inactivated BEFV vaccine. At 3 weeks after initial vaccination, the cattle received a second immunization at the same dose. Blood was collected 3 weeks after the first inoculation and 2 weeks after the second.

### Serum neutralizing antibody titration

For the neutralization assay, sera were heat-inactivated at 56 °C for 30 min. Serial twofold dilutions were mixed with equal volumes of virus diluted to contain 100 TCID_50_/50 μl BEFV. The mixture was incubated for 1 h at 37 °C in 5 % CO_2_. Then, 100 μl of the serum–virus mixture was transferred to BHK-21 cell monolayers in 96-well plates and incubated for 1 h at 37 °C. The monolayers were added to 100 μl Dulbecco’s modified Eagle’s medium. After incubation for 72 h at 37 °C, a cytopathic effect was observed. Neutralization titers were expressed as the reciprocal of the highest dilution of serum that resulted in at least a 50 % reduction in the number of infected cells relative to the negative control. This assay was performed as described previously [9].

### Statistical analysis

Data on virus and antibody titers were analyzed by Student’s *t*-test using the Excel program (Microsoft, Redmond WA, USA).

## Results

### Expression of BEFV G protein by rL-BEFV-G

**Fig. 1:**
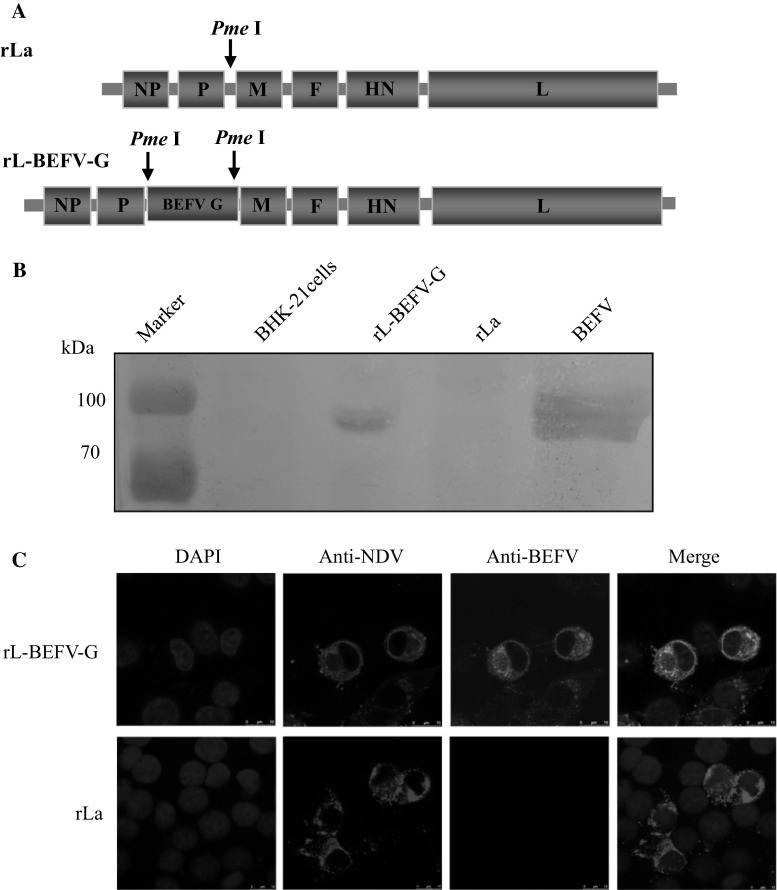
Construction and identification of rL-BEFV-G. (A) Schematic representation of the rLaSota genome and BEFV G inserted between the P and M genes. (B) Western blot demonstrating the expression of BEFV G. BHK-21 cells were infected with rLaSota or rL-BEFV-G at an MOI of 1. After 24 h, cells were collected and lysed, and proteins in the cell lysate were separated by SDS-PAGE and immunoblotted with mouse anti-NDV antibodies or BEFV G polyclonal antibody. (C) Immunofluorescence analysis of BEFV G protein expression. BHK-21 cells were infected with rLaSota or rL-BEFV-G at an MOI of 0.01. After 24 h, the cells were fixed and then stained with chicken anti-NDV antibody or BEFV G polyclonal antibody, followed by incubation with FITC-conjugated goat anti-mouse antibody or TRITC-conjugated rabbit anti-chicken antibody

The BEFV G gene ORF was cloned between the P and M genes of the NDV genome at the PmeI site, using the NDV LaSota virus reverse genetic system established by Ge et al. [12–15] in which a unique PmeI site was introduced between the P and M gene when constructing a full-length NDV genome plasmid (Fig. 1A). The recombinant virus rL-BEFV-G was recovered entirely from this cDNA using established reverse genetics procedures [13, 30]. To detect expression of BEFV G, BHK-21 cells were infected with rL-BEFV-G at an MOI of 1. Total proteins from cells infected with rL-BEFV-G or rLaSota were analyzed by western blotting using antibodies against BEFV. Western blotting demonstrated that rL-BEFV-G reacted with antibodies against BEFV from mice, producing a band of ~80 kDa, which is equal to the molecular mass of BEFV G. However, the vector rLaSota did not react with the anti-BEFV antibodies, and no band was detected (Fig. 1B). BHK-21 cells were also infected with rL-BEFV-G at an MOI of 0.01, and at 48 h after infection, the cells were fixed and incubated with antibodies against BEFV, followed by FITC-conjugated goat anti-mouse antibody or TRITC-conjugated rabbit anti-chicken antibody. Confocal immunofluorescence showed that BEFV G was expressed in cells infected with recombinant virus (Fig. 1C). These results confirmed that BEFV G could be correctly expressed from recombinant rL-BEFV-G.

### BEFV G expression enables rL-BEFV-G to spread from cell to cell

**Fig. 2:**
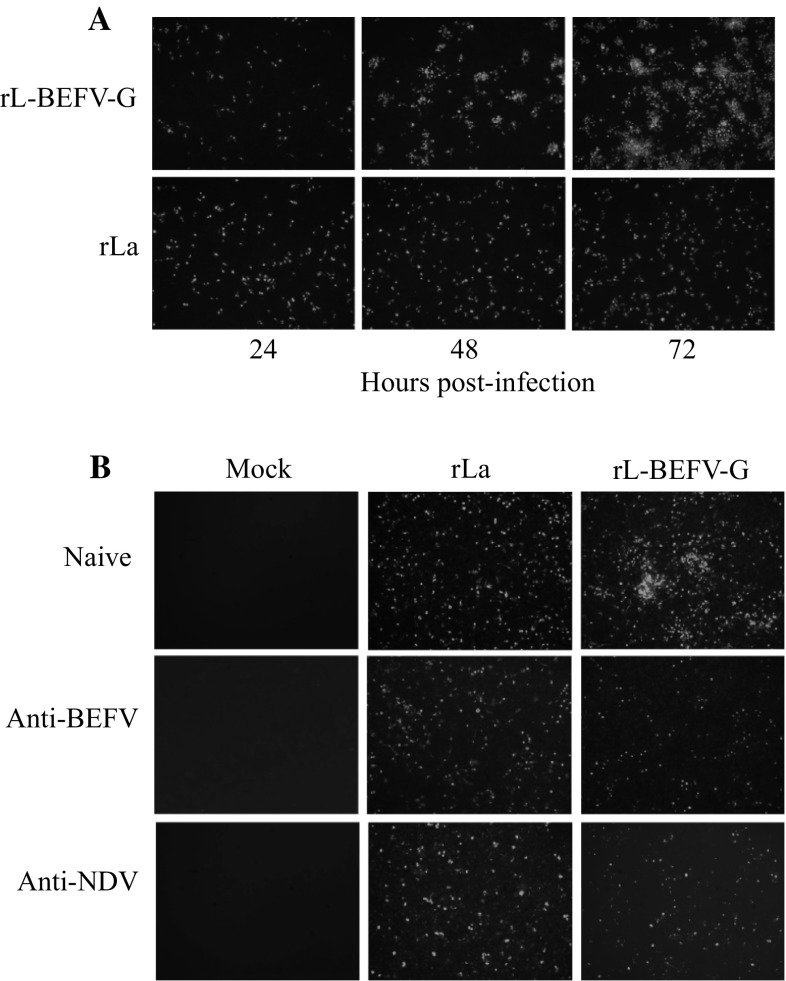
Cell-to-cell spread of rL-BEFV-G and NDV vector in BHK-21 cells. (A) Monolayers of BHK-21 cells were infected with either rLaSoTa or rL-BEFV-G at an MOI of 0.05. After five washes 1 h postinfection, the infected cells were incubated at 37 °C. The infected cells were examined at the indicated times post-infection using IFA with chicken serum against NDV. (B) Assay for inhibition of intercellular spread of recombinant virus in BHK-21 cells. Monolayers of BHK-21 cells were infected with either rL or rL-BEFV-G at an MOI of 0.05. After five washes 1 h postinfection, the cells were incubated with culture medium containing 100-fold-diluted mouse serum against NDV, mouse serum against BEFV, or naïve mouse serum. Infected cells were examined 72 h postinfection using IFA with chicken serum against NDV

BHK-21 cells were infected with rLaSota or rL-BEFV-G at an MOI of 0.05. At different times post-inoculation, cells were fixed and stained with fluorescein. rLaSota was observed to infect individual cells, but the infection did not spread to adjacent cells. At 24 h, cell-to-cell spread was observed in cells infected with rL-BEFV-G (Fig. 2A). At 72 h, fluorescent plaques caused by intercellular spread of virus were observed (Fig. 2A). BEFV and NDV serum antibody could block the intercellular spread of recombinant virus (Fig. 2B). These results suggest that BEFV G enables rLaSota to spread from the initial infected cell to adjacent cells.

### Expression of the BEFV G gene does not increase the virulence of the NDV vector in poultry

**Fig. 3:**
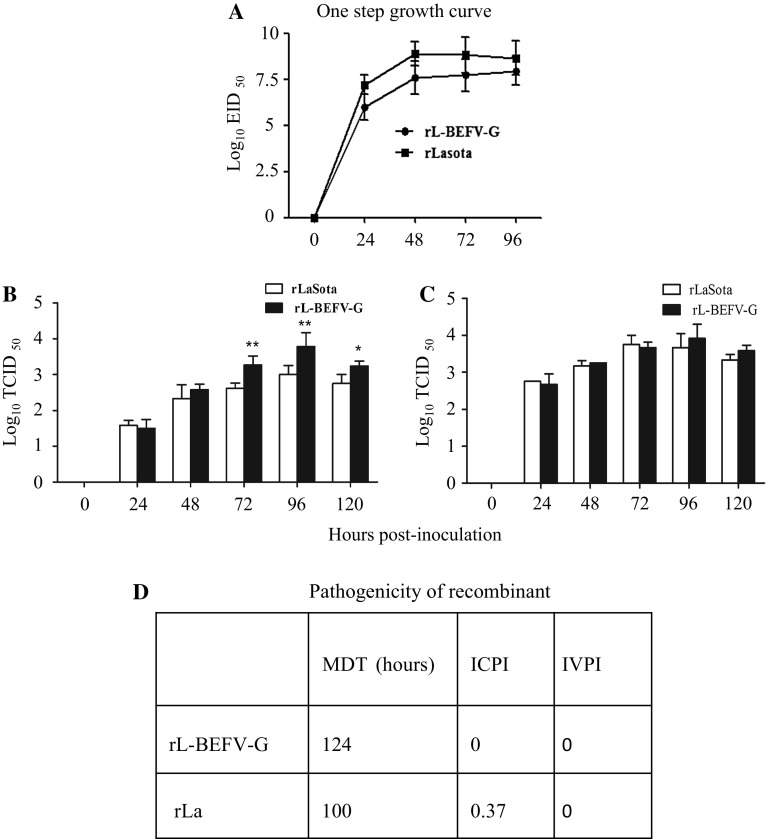
Biological characteristics of rL-BEFV-G in poultry. (A) Kinetics of rL-BEFV-G replication in embryonated eggs. Ten-day-old embryonated eggs were infected with rLa or rL-BEFV-G at 10^5^ TCID_50_. Allantoic fluid of six eggs from each group was harvested at 24, 48, 72 and 96 h postinoculation, and virus titers were determined in EID_50_ units in 10-day-old embryonated eggs. (B) Kinetics of rL-BEFV-G replication in MDBK cells. Monolayers of MDBK cells were infected with either egg-propagated rLaSota or rL-BEFV-G at an MOI of 0.01. After five washes 1 h postinfection, the cells were incubated with 100-fold-diluted mouse serum against NDV for 30 min to neutralize the residual viruses in the supernatants. After replacement of the medium with fresh medium, the infected cells were incubated at 37 °C in the absence (B) or presence (C) of TPCK trypsin (1 μg/ml). The culture supernatants were collected at different times, and their virus was titrated in MDBK cells with 1 μg of TPCK trypsin per ml. Significant differences between rLa and rL-BEFV-G were observed using Student’s *t*-test. *, P <0.05; **, P < 0.01. (D) Pathogenicity assay in SPF eggs and chickens. MDT, ICPI and IVPI were determined according to the recommended OIE method

Growth kinetics were analyzed in chick embryos and MDBK cells. The replication of rL-BEFV-G was similar to that of vector rLaSota in chick embryos and MDBK cells (Fig. 3). However, in the absence of TPCK trypsin, the rL-BEFV-G titers were higher than those of rLaSota at same time point (Fig. 3B).

To determine whether BEFV G expression influenced the virulence of rLaSoTa, the MDT, ICPI and IVPI values were tested generically as parameters for evaluating the pathogenicity of NDV strains in poultry [13, 18]. Strains of NDV were categorized into three groups on the basis of their MDT (velogenic, <60 h; mesogenic, 60–90 h; and lentogenic, >90 h and ICPI: velogenic, >1.60; mesogenic, 1.20–1.60; lentogenic, <1.20 values) [1, 31]. The values of MDT for rLaSota and rL-BEFV-G were 100 and 124 h, respectively (Fig. 3D). The ICPI values for rLaSota and rL-BEFV-G were 0.37 and 0, respectively (Fig. 3D). The IVPI values for rLa and rL-BEFV-G were both 0 (Fig. 3D).

### Expression of BEFV G gene does not increase the virulence of the NDV vector in mice

**Fig. 4:**
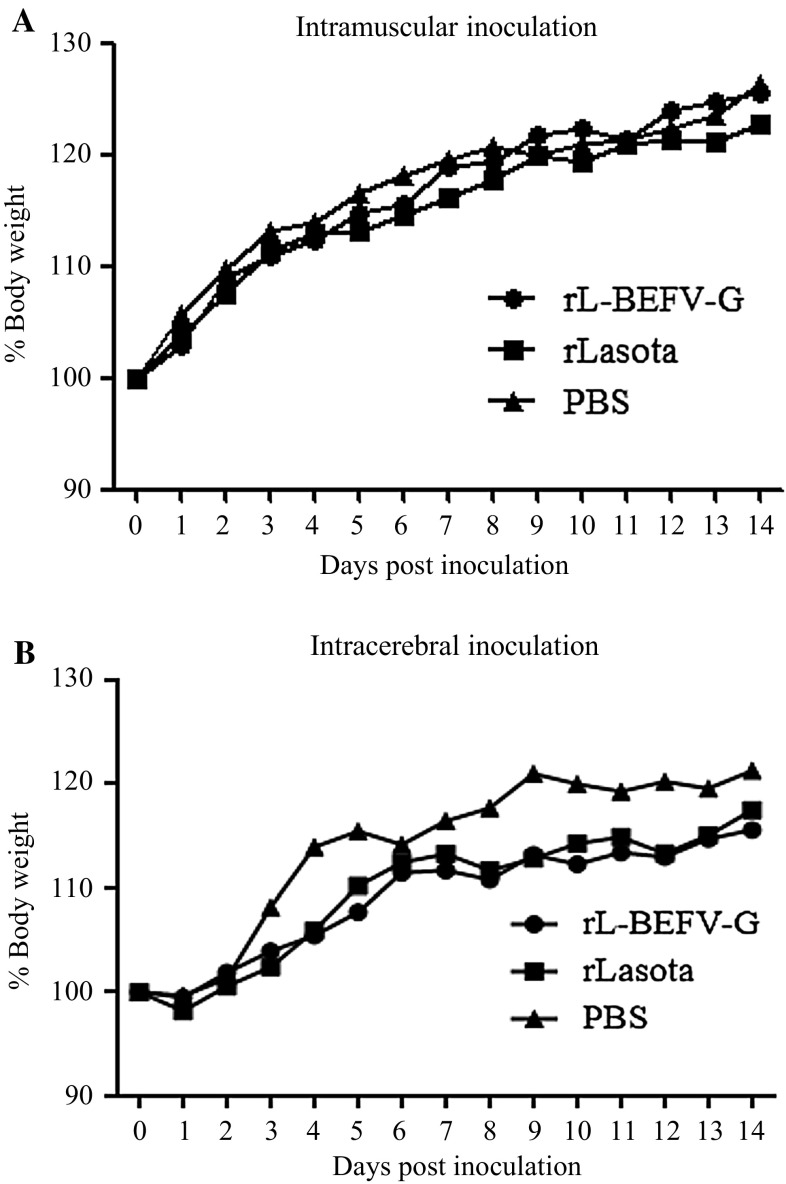
Changes in body weight in mice inoculated with rL-BEFV-G. Mice were inoculated intramuscularly (A) or intracerebrally (B) with 10^7^ TCID_50_ of rL-BEFV-G on day 0. Mice were observed and weighed daily from day 0 to 14. All mice survived for the duration of the experiment. Body weight changes for each group are shown as ratios relative to the body weight at day 0, which was set as 100 %

To investigate the pathogenicity of the recombinant virus in mammals, mice were inoculated intracerebrally and intramuscularly with rLaSota and rL-BEFV-G, respectively. All of the mice survived after inoculation. There were no differences between rLaSota and rL-BEFV-G infection in terms of body weight changes after intramuscular (Fig. 4A) or intracerebral (Fig. 4B) inoculation, and no clinical symptoms were observed. Virus was not detected by IFA or PCR in any of the organs (data not shown).

### rL-BEFV-G induces an immune response in mice

**Fig. 5:**
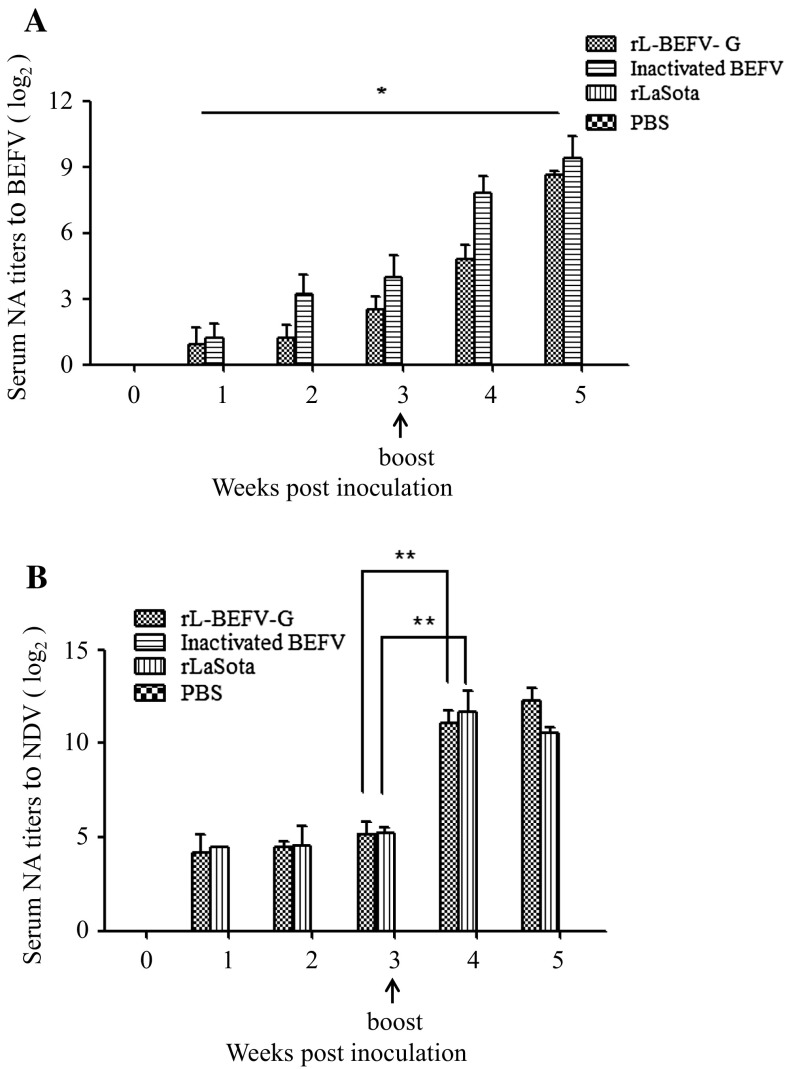
Serum neutralization analysis in mice. Serum samples were collected at different times after vaccination, and SN antibody titers for BEFV (A) and NDV (B) were measured. Statistically significant differences were determined using Student’s *t*-test. *, P < 0.05

Forty mice were inoculated with rL-BEFV, rLaSota, inactivated BEFV vaccine and PBS. Both rL-BEFV-G and the inactivated BEFV vaccine induced an immune response after inoculation. At 3 weeks after immunization, the titer of the serum neutralizing (SN) antibodies against BEFV was 1:6 in the rL-BEFV-G group and 1:16 in the inactivated BEFV group (Fig. 5A). At 2 weeks after booster immunization, the SN antibody titer was significantly increased in the rL-BEFV-G and inactivated BEFV groups. The SN antibody titer was 1:388 in the rL-BEFV-G group and 1:676 in the inactivated BEFV group. The SN antibody titers for NDV were similar for the rL-BEFV-G and rLaSota groups (Fig. 5B).

### rL-BEFV-G induces an immune response in cattle

Eight 1-year-old BEFV-seronegative Holstein calves were allotted randomly to the rL-BEFV-G and inactivated-BEFV-vaccine groups. The cattle in the rL-BEFV group were immunized with 4 ml of allantoic fluid with 2 × 10^7^ TCID_50_ by intramuscular injection. The cattle in the inactivated BEFV vaccine group were immunized with commercial inactivated vaccine by the same route of administration. The SN antibodies in the rL-BEFV-G and inactivated BEFV groups were titrated after the first and second immmunizations. After the first dose, most cattle produced detectable SN antibody (Table 1). After the second immunization, the SN antibody titer was significantly increased (Table 1). Generally, the commercial inactivated vaccine induced higher SN antibody titers than did rL-BEFV-G. rL-BEFV-G induced a protective effect level of SN antibody (In the field experiments, the titer was 1:32, which could provide protection.)

**Table 1:** Immune effect in cattle

Vaccines	Calf no.							
	1	2	3	4	1	2	3	4
Inactivated BEFV vaccine	1:11^a^	1:10^a^	1:23^a^	1:7^a^	1:512^b^	1:1722^b^	1:861^b^	1:1024^b^
rL-BEFV-G	1:8^a^	1:16^a^	<1:8^a^	1:8^a^	1:64^b^	1:128^b^	1:64^b^	1:128^b^

^a^3 weeks post the first immunization

^b^3 weeks post the second immunization

## Discussion

BEFV causes an acute febrile infection in cattle and water buffalo [40] and often results in heavy economic losses [6]. To date, there have been few reports about BEFV vaccines. The safety and efficacy of NDV as a viral vector has been evaluated in many animals, such as African green monkeys, rhesus monkeys, pigs, mice, cattle, and chickens, as well as in humans [3, 8, 12, 13, 15, 16, 18, 22]. Here, we used reverse genetics to generate a recombinant NDV, rL-BEFV-G, that expresses the BEFV glycoprotein. We demonstrated that BEFV G was correctly expressed in BHK-21 cells infected with rL-BEFV-G. To evaluate safety, poultry and mice were infected with rL-BEFV-G. BEFV G inserted into NDV rLaSota did not change its lentogenic nature. In this study, all of the results demonstrated that the use of NDV as a virus vector was safe in mice, as reported previously [9, 12, 13, 21].

Viruses can spread by two fundamentally distinct modes, either by diffusion through the extracellular space or by direct cell–cell contact [28, 35, 45]. NDV cannot spread by direct cell–cell contact in BHK-21 cells without trypsin. However, BEFV G expression changed NDV transmission in BHK-21 cells, and the NDV vector acquired the ability to spread among BHK-21 cells. The rLRVG could not blocked by antibody against NDV [13], but in this study, when we added an anti-NDV serum, the ability of rL-BEFV-G to spread from cell to cell was abolished. The mechanism by which this occurs will be explored in the future. In the case of other viruses, such as herpes simplex virus, the transmembrane (TM) or cytoplasmic (CT) domains of gE and gI are essential for epithelial cell-to-cell spread, which relies on both the CT domains of gE/gI, which sort the virus to cell junctions, and the extracellular domains, which function to promote entry into other host cells [27]. The mechanism of intercellular spread of rL-BEFV-G will be investigated further.

rL-BEFV-G induced a good immune response in mice and cattle. The titers were 1:388 and 1:64–128, respectively. Other live-vector vaccines for BEFV have been reported. Vaccinia virus expressing BEFV G induced neutralizing antibody with a titer of ~1:100 after the second inoculation and provided protection against experimental BEFV infection in cattle [17]. BEFV G vectored by the South African vaccine strain of lumpy skin disease virus- could induce neutralizing antibody and cellular immune responses, but gave unsatisfactory protection from virus challenge [41]. In this study, the SN antibody titer was induced by the replication-defective NDV vector 1:128 in cattle. Additionally, rL-BEFV-G has the advantage that it is easy to culture and grow to high titers in chicken eggs; a high titer (256 to 512) of SN antibody can be obtained by increasing the inoculation dose [13], and a high concentration of the virus can be obtained from allantoic fluid. Additionally, NDV, as a live viral vector, can induce cellular immunity [18, 32] and be used to distinguish the wild-type virus from the vaccine strain.

As a result of the instability of BEFV, it was difficult to carefully regulate the challenge dose prior to the trial and successfully duplicate clinical symptoms. In this study, we did not perform a challenge test, but this needs to be done in the future.

In conclusion, our results demonstrate that rL-BEFV-G is safe in mice and chickens. rL-BEFV-G induces high levels of neutralizing antibodies in mice and cattle, and thus probably confers good protection against BEFV challenge. rL-BEFV-G appears to be a promising candidate vaccine against BEFV.
